# Quality Evaluation of Agricultural Distillates Using an Electronic Nose

**DOI:** 10.3390/s131215954

**Published:** 2013-11-25

**Authors:** Tomasz Dymerski, Jacek Gębicki, Waldemar Wardencki, Jacek Namieśnik

**Affiliations:** 1 Department of Analytical Chemistry, Chemical Faculty, Gdansk University of Technology, 11/12 G. Narutowicza Str., 80-233 Gdańsk, Poland; E-Mails: waldemar.wardencki@pg.gda.pl (W.W.); jacek.namiesnik@pg.gda.pl (J.N.); 2 Department of Chemical and Process Engineering, Chemical Faculty, Gdansk University of Technology, 11/12 G. Narutowicza Str., 80-233 Gdańsk, Poland; E-Mail: jacek.gebicki@pg.gda.pl

**Keywords:** electronic nose, agricultural distillates, PCA, cluster analysis

## Abstract

The paper presents the application of an electronic nose instrument to fast evaluation of agricultural distillates differing in quality. The investigations were carried out using a prototype of electronic nose equipped with a set of six semiconductor sensors by FIGARO Co., an electronic circuit converting signal into digital form and a set of thermostats able to provide gradient temperature characteristics to a gas mixture. A volatile fraction of the agricultural distillate samples differing in quality was obtained by barbotage. Interpretation of the results involved three data analysis techniques: principal component analysis, single-linkage cluster analysis and cluster analysis with spheres method. The investigations prove the usefulness of the presented technique in the quality control of agricultural distillates. Optimum measurements conditions were also defined, including volumetric flow rate of carrier gas (15 L/h), thermostat temperature during the barbotage process (15 °C) and time of sensor signal acquisition from the onset of the barbotage process (60 s).

## 1. Introduction

The quality of agricultural distillates depend on their composition and due to their complexity some problems can be encountered during their purification (rectification). Thus, an important stage of rectified alcohol production is the quality control and classification of the agricultural distillates [1]. In a number of industrial alcohol plants quality control and classification of raw material is done using organoleptic analysis characterized by many shortcomings originating from the limitations of the human senses. As a consequence, alcohol industry representatives report that distillery plants collect agricultural distillates of low quality. In fact there are still no unequivocal methods of differentiation between high quality agricultural distillates and those produced from worse quality raw materials or using improper technology. Processing of raw alcohol of low quality results in high costs for further processing, causing financial losses to the manufacturer.

Currently recognized manufacturers of alcohols tend to label their products in a detailed way following the requirements of the European Union, which are contained in the Regulation (EC) No. 110/2008 of the European Parliament and of the Council of 15 January 2008 on the definition, description, presentation, labeling and the protection of geographical indications of spirit drinks and repealing Council Regulation (EEC) No. 1576/89. In this way the manufacturers want to assure potential clients that their products are of the highest quality and are produced from the finest, single raw materials. Sensory quality is primarily driven by the volatile organic compounds contained in a volatile fraction of the agricultural distillates. Quality and type of starch and other raw materials as well as hygiene and conditions of the fermentation and distillation process influence substantially the aroma of the raw alcohol supplied to the distillery plant. Earlier studies reveal that utilization of non-rectified ethyl alcohol in industrial conditions yields a number of by-products: carbonyl compounds, alcohols, esters, acids and acetals [1]. These compounds are present in a very wide concentration range. Some of them have a beneficial influence on sensory features (for instance esters), while others impart a negative impact even when present in only trace amounts (for example aldehydes).

Application of electronic nose instruments in the alcohol industry has already been investigated [2–11]. However, the literature does not report on analysis of agricultural distillates using electronic noses as far as their quality is concerned. What is most frequently described is testing of final alcohol products—whisky, brandy, vodka, wine, beer—their classification, storage conditions, degree of ripening of the fruits they are made of as well as their botanic and geographic origin [3,8,11–20]. Moreover, the application of electronic noses can substantially reduce the time necessary for a single analysis of the raw material supplied to a distillery plant. This approach has an advantage over chromatographic techniques (frequently utilized in industrial investigations of agricultural distillate quality). A single gas chromatography analysis takes about 30–50 min [21–34], whereas a typical electronic nose analysis lasts a few minutes [19,35–38]. This difference can be a milestone in industrial analytics of raw alcohol.

This paper presents our investigations using an electronic nose prototype of equipped with six low-cost, semiconductor sensors by FIGARO Co. (Osaka, Japan). The aim of the research was to verify the usefulness of this device in differentiating between high class agricultural distillates fulfilling the requirements of the Polish standard and medium and low class ones. The intention of the authors was also to verify whether this type of device, dedicated exclusively to agricultural distillate evaluation and several times cheaper than commercially available electronic nose devices, is capable of finding future application in the industry. Moreover, the authors hope the presented results could contribute in the future to the wider application and development of fast, non-invasive electronic nose techniques in the field of industrial investigations of alcohol products.

## 2. Experimental Section

### 2.1. Measurement Set-Up

**Figure 1.**
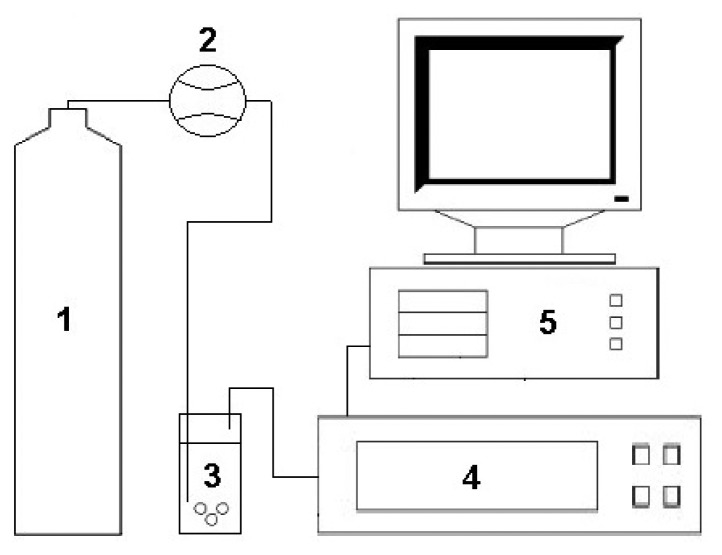
Experimental set-up for analysis of volatile fraction of agricultural distillates consisting of: 1—bottle with carrier gas; 2—flow meter; 3—scrubber; 4—prototype of electronic nose; 5—PC.

The measurement set-up consisted of a bottle with carrier gas, a Tecfluid 2150 series flow meter (Tecfluid S.A., Barcelona, Spain), an electronic nose prototype and a PC class computer. Compressed air of N5.0 purity (Linde Gaz Polska Ltd., Gdańsk, Poland) was the carrier gas. All elements of the measurement set-up, from the bottle with carrier gas to the prototype of electronic nose, were connected with Teflon tubes of 4 mm diameter. The measurement set-up also contained a bottle reducer with a metal membrane dedicated for non-corrosive gases (IHW Group, Berlin, Germany) mounted in order to provide a chemically inert environment for the carrier gas all the way from the bottle to the electronic nose. The above-mentioned measurement set-up has already been described in the literature [35] and is presented in Figure 1.

### 2.2. Electronic Nose Prototype

The prototype consisted of a set of six commercial semiconductor sensors by FIGARO Co. (TGS 880, TGS 825, TGS 826, TGS 822, TGS 2610, TGS 2602). Relative humidity of the gas mixture flowing through a module with the sensors depended on the thermostats' temperature as well as the volumetric flow rate of the carrier gas and ranged from 70% to 90%. A dedicated miniaturized electronic circuit conditioned the output signal from the sensor set of the prototype. Application of this circuit enabled lowering the limit of detection of the semiconductor sensors by one order of magnitude. Utilization of a set of thermostating modules providing gradient temperature characteristics to the investigated gas mixtures obtained via a barbotage process [39] made it possible to acquire stable, reproducible sensor response signals. Four thermostating modules contained within the electronic nose prototype allowed for a constant temperature gradient preventing condensation of the gaseous agricultural distillate samples of all the way from the sample chamber to the exit from the electronic nose system. It enabled performing a number of successive measurements (eliminating the so-called ‘wall memory effect’). Reproducibility of 93% of the results obtained from the e-nose prototype was within 5.8%–9.2% *Coefficient of Variation* (CV). In order to calculate CV coefficients five analyses were conducted for a sample of distillate (each point in the multidimensional data space subjected to further statistical analysis was a mean of five measurement points).

### 2.3. Software for Chemometric Data Analysis

Chemometric data analysis was performed with the commercially available software SAS Enterprise 4.3 (SAS Co., Cary, NC, USA) with the implemented PRINCOMP algorithm (for principal component analysis) and STATISTICA 10 (StatSoft Co., Tulsa, OK, USA) software (for the single-linkage cluster analysis and for the spheres method).

### 2.4. Object of Investigation

The investigations were conducted on agricultural distillates of different raw material origin: triticale, corn, wheat, barley, bread rye, diamond rye and golden rye. The agricultural distillate samples originated from local distilleries of the Pomeranian voivodship. The results of organoleptic evaluations performed in a laboratory of Sobieski Distillery S.A. in Starogard Gdański (Poland) allowed classification of the agricultural distillates into three quality classes: high quality class: six distillates, which earned very high marks during sensory analysis according to the Polish standard PN-A-79528-2:2002, medium quality class: six distillates, for which there was a discrepancy between testers during the sensory analysis—some testers classified them as compliant with the standard, some rejected them as incompliant, low quality class: six distillates, which were unanimously classified as incompliant.

### 2.5. Preparation of Samples for Analysis

Preparation of the agricultural distillate samples consisted in dilution with deionized water (Mili-Q A10 device by Millipore Co., Billerica, MA, USA) in 1:1,000 ratio. Then 5 mL of diluted sample was collected and transferred to a 15 mL vial. The total number of prepared samples was 990. The investigations were performed over 2 months.

## 3. Results and Discussion

**Figure 2.**
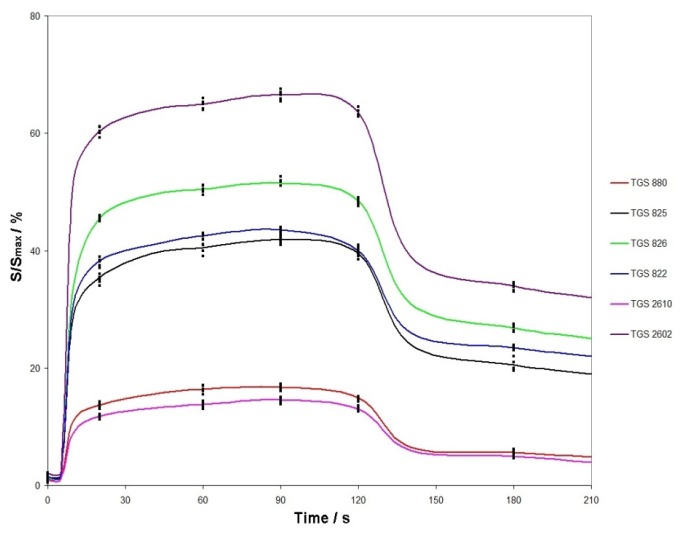
TGS sensors response signal *versus* time. Volumetric flow rate of carrier gas −15 L/h, thermostating temperature of sample subjected to barbotage process 15 °C.

Figure 2 shows exemplary response signals of the sensors *versus* time. They are presented as a ratio of the signal (expressed in bits, analogue-to-digital converter changed the voltage signal into digital one) to the maximum signal (a complete range of changes of digital signal, which was 14 bits). For each dependency between sensor response signal and time, the response signal was recorded at selected time instants of 20, 60, 90, 120 and 180 s. These measurements were repeated four times for every sample of a particular quality class at a given volumetric flow rate of carrier gas and for a given thermostating temperature of the sample subjected to the barbotage process. Colour lines designate the averaged (over five measurements) dependence between sensor signals and time. Data analysis using PCA and cluster analysis was performed for selected time instants on averaged sensor characteristic signals.

In order to assess optimum barbotage conditions and optimum operation parameters of the electronic nose a series of investigations was carried out aimed at determination of the influence of barbotage process temperature, carrier gas volumetric flow rate and time of sensor signal acquisition since the onset of the barbotage process.

### 3.1. Investigation of the Barbotage Temperature Influence

**Figure 3.**
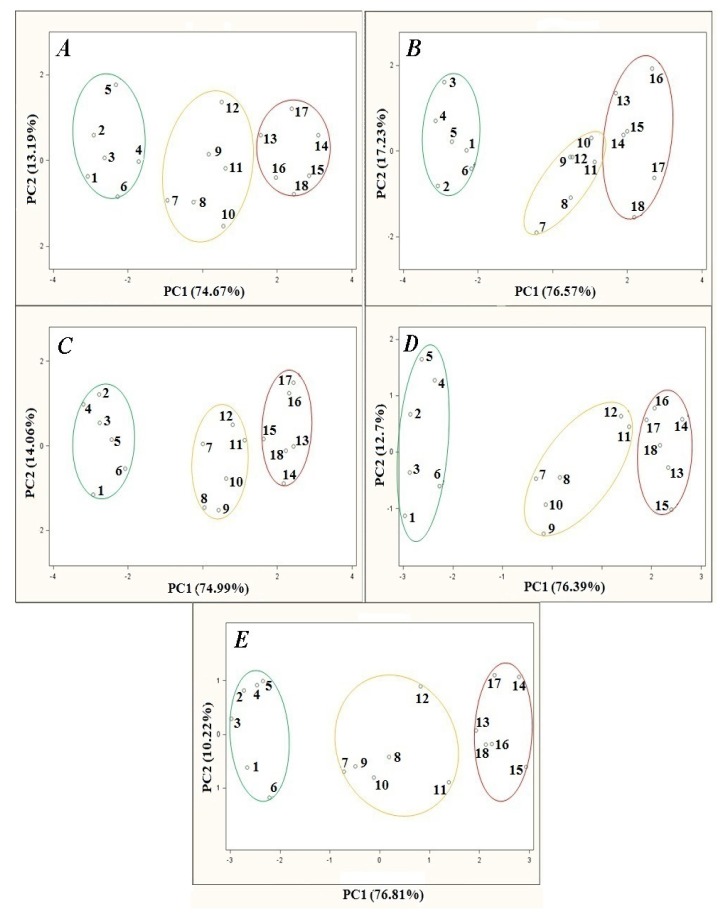
PCA results for samples of agricultural distillates obtained for different barbotage temperatures: (**A**) 35 °C; (**B**) 30 °C; (**C**) 25 °C; (**D**) 20 °C; (**E**) 15 °C; points: 1–6—high quality class distillates (green), 7–12—medium quality class distillates (yellow), 13–18—low quality class distillates (red).

Investigation of the temperature influence on the barbotage process consisted in changes of the temperature of a heating jacket with the samples inside and their subsequent analysis. The temperature influence was investigated within a 15 °C–35 °C range with 5 °C steps. Figure 3 presents the results of principal component analysis of the agricultural distillates *versus* barbotage temperature changes. The remaining parameters of the barbotage process and electronic nose operation were kept constant during every analysis and were as follows: sample volume 5 mL, volumetric flow rate of carrier gas 15 L/h, time of sensor signal acquisition since barbotage process onset 60 s. Looking at the PCA results in Figure 3 one can notice that as the barbotage temperature decreases from 35 °C to 15 °C in 5 °C steps there is an improvement in the separation of the points group associated with high class agricultural distillates from the medium and low class points on the PC1PC2 plane. Analyzing the points groups representing medium and low class distillates only, the following dependences can be found: the smallest separation of the points groups is in the case of the PCA results presented in Figure 3A and 3B, relatively larger separation of the points groups occurs for the PCA results shown in Figure 3C and Figure 3D as compared to the PCA results illustrated in Figure 3A and 3B. A separation of the points groups in case of the PCA results presented in Figure 3C and Figure 3D is similar to the PCA results in Figure 3E, however the distance of the point no. 11 and the point no. 12 from the nearest points representing low class distillates is the largest for the analysis result presented in Figure 3E. The PCA result presented in Figure 3E has been concluded the best as far as separation of the points groups is concerned.

### 3.2. Investigation of Volumetric Flow Rate Influence

**Figure 4.**
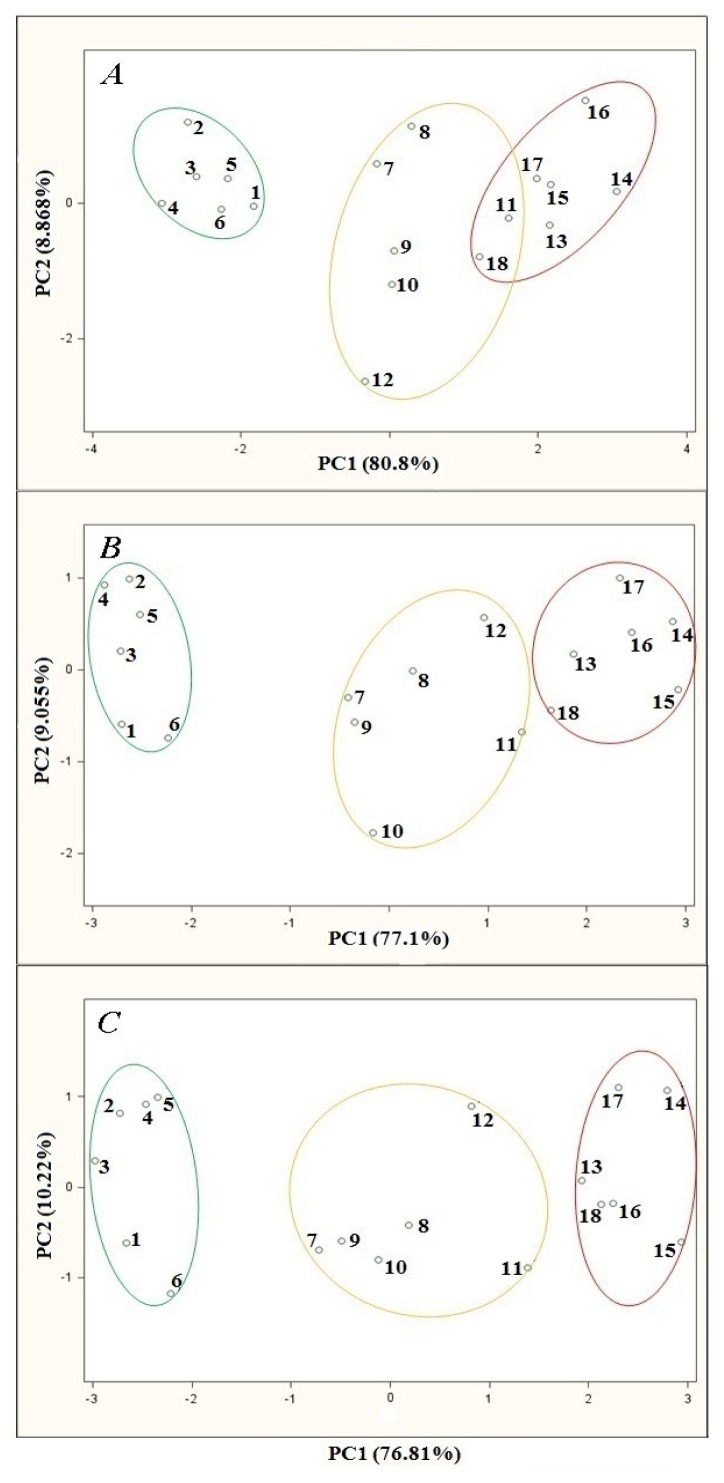
PCA results for samples of agricultural distillates obtained for different volumetric flow rate: (**A**) 5 L/h; (**B**) 10 L/h; (**C**) 15 L/h; points: 1–6—high quality class distillates (green), 7–12—medium quality class distillates (yellow), 13–18—low quality class distillates (red).

Investigation was carried out for the following volumetric flow rates: 5, 10 and 15 L/h. The remaining parameters of the barbotage process and electronic nose operation were kept constant during every analysis and were as follows: sample volume 5 mL, barbotage temperature 15 °C, time of sensor signal acquisition since barbotage process onset 60 s. PCA results showing the influence of volumetric flow rate of carrier gas are presented in Figure 4. It can be seen on the PCA plot that for the flow rates of 5, 10 and 15 L/h the points group representing high class distillates is relatively distant from the points groups of medium and low class distillates, which allows differentiation of the high class products from the remaining ones.

In the case of analysis for the carrier gas volumetric flow rate of 5 L/h there is an overlap of the regions engulfing the points groups representing medium and low class distillates. A change of volumetric flow rate to 10 L/h results in a separation of these two points groups, nevertheless they remain relatively close to each other on the PCA plot. There is particularly small distance between the point no. 11 (belonging to a medium class distillate) and the point no. 18 (assigned to a low class distillate). This distance is smaller than some of the distances between the points belonging to the same group. An increase in the carrier gas volumetric flow rate to 15 L/h results in an increase in the distance between these two groups of points. The coordinates of the point no. 11 (the point closest to the neighboring points group) differ substantially from the coordinates of the points belonging to low class distillates. The dependences described above can be explained by the fact that for higher volumetric flow rate there is better dispersion of the carrier gas in liquid sample and more effective transfer of the analytes from liquid to gas phase during barbotage.

### 3.3. Determination of the Optimum Time of Sensor Signal Acquisition

**Figure 5.**
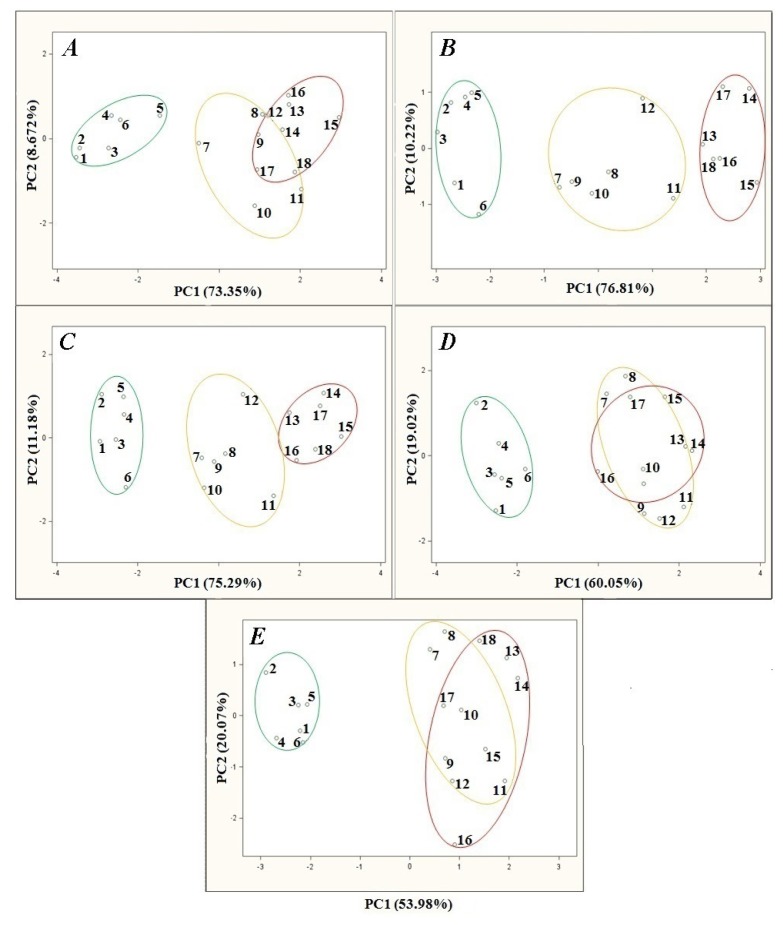
PCA results of samples of agricultural distillates for different time of sensor signal acquisition since barbotage process onset: (**A**) 20 s; (**B**) 60 s; (**C**) 90 s; (**D**) 120 s; (**E**) 180 s; points: 1–6—high quality class distillates (green), 7–12—medium quality class distillates (yellow), 13–18—low quality class distillates (red).

Figure 5 presents the PCA results for the samples of agricultural distillates depending on time of sensor signal acquisition since the onset of the barbotage process. Also in this case the remaining parameters of the barbotage process and electronic nose operation were kept constant during every analysis and were as follows: sample volume 5 mL, volumetric flow rate of carrier gas 15 L/h, barbotage temperature 15 °C.

Figure 5A presents the PCA results obtained when the measurement started 20 s after barbotage onset. Such a short time enabled the separation of the points group belonging to the high class distillates from the points groups representing medium and low quality distillates. On the other hand a distance between the point no. 5 and the closest neighboring point of high class distillates is comparable to the distance between the point no. 5 and the closest point belonging to the medium class distillates group (point no. 7). Moreover, the regions of the points representing medium and low class distillates overlap. Accordingly, proper classification of the samples of medium and low class distillates is not possible and classification of high quality agricultural distillates is burdened with relatively high uncertainty. Such a situation can be caused by insufficient extraction of the analytes from the liquid to the gas phase, so the profiles of the volatile fractions are less differentiated. Elongation of the time of sensor signal acquisition to 60 s makes complete differentiation of the aforementioned points groups possible. Additionally, the profiles of the volatile fractions are more differentiated than in the previous case. For the following PCA results presented in Figure 5C–E there is a deterioration of the points groups separation. In case of the PCA results presented in Figure 5D and 5E the regions of points representing medium and low class distillates overlap. The reason could be the fact that for the time of sensor signal acquisition equal or greater than 120 s since the barbotage onset there was earlier that extraction of more volatile analytes from the sample, so the volatile compounds profiles were less differentiated.

### 3.4. Final Outcome of the Optimization Investigations

**Figure 6.**
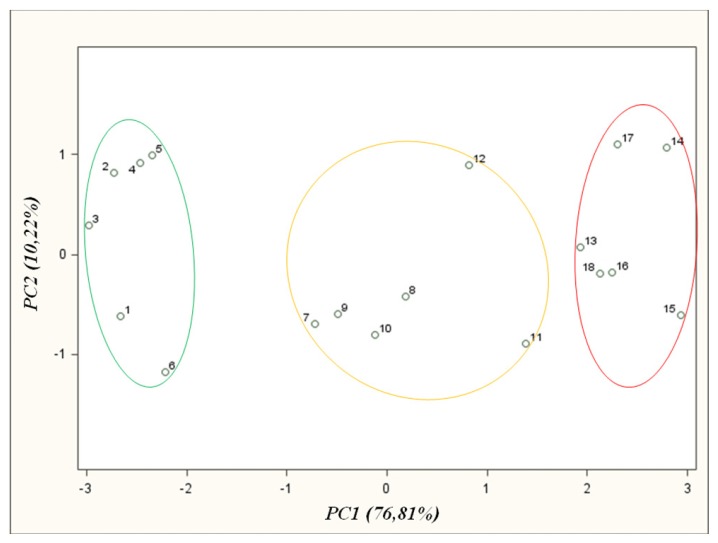
PCA results for samples of agricultural distillates obtained for optimum conditions of barbotage process and optimum operation parameters of electronic nose prototype; points: 1–6—high quality class distillates (green), 7–12—medium quality class distillates (yellow), 13–18—low quality class distillates (red).

Figure 6 illustrates the final PCA result, which was already presented earlier together with other results in the previous chapters of this paper, characterized by the best separation of three groups of points.

The distances between particular groups are big enough to differentiate between the points belonging to given groups. This result enables differentiation of high class agricultural distillates from medium and low quality ones.

**Figure 7.**
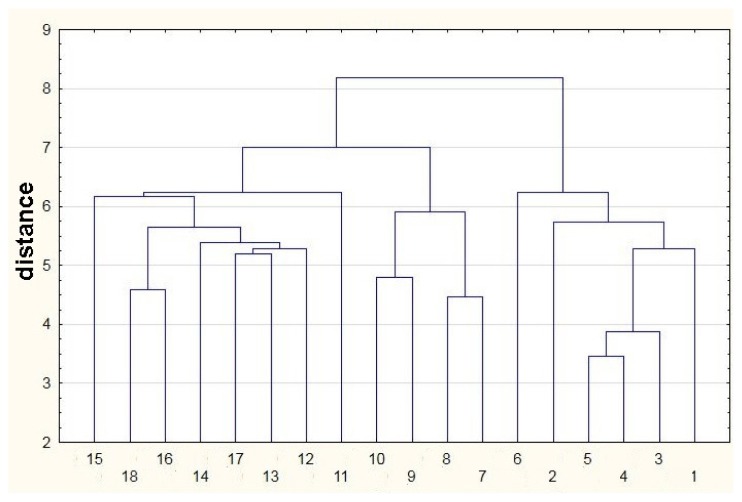
Cluster diagram of single-linkage (nearest neighbor) clusters for samples of agricultural distillates; points: 1–6—high quality class distillates (green), 7–12—medium quality class distillates (yellow), 13–18—low quality class distillates (red).

Figure 7 presents the cluster diagram obtained using single-linkage cluster analysis. A measure of distance between objects is the Euclidean distance. In this method a distance between two clusters is defined by a distance between two closest objects (the nearest neighbors) belonging to different clusters. Following this rule the objects form clusters by combining into series and the resultant clusters form long ‘chains’. Cluster analysis belongs to a group of graphical representations of information contained in a distance matrix. This method presents hierarchical grouping of the objects in the form of a suitable diagram (tree). The horizontal axis of the diagram is completely arbitrary and does not possess the character of number axis. On the vertical axis one denotes distance or probability, for which two objects create a cluster. Looking at the diagram in Figure 7 it can be stated that the analyzed features are divided into three clear clusters. The first one engulfs the features from 1 to 6, the second one gathers the features from 7 to 10 and the third one contains the features from 11 to 18. This analysis confirms an ability to differentiate high class agricultural distillates from the remaining ones.

Another method of cluster analysis is a direct method called the spheres method. It is based on an assumption that the objects within a group focus around certain central point. The first step of analysis is a selection of the object within the group, which is the closest to the central point. Inside a hypersphere of radius R, for every object one calculates a number of other objects located within R distance from the selected object. The radius R is determined using the following relation:

(1) $$R=\max\left|\min(d_{ij})\right|$$

where: *d_ij_* is the smallest Euclidean distance between the objects *i,j* = *1*,…,*n*.

The analysis with spheres method yields that the features 1–6 belong to the first sphere, 7–10 belong to the second sphere, 11–13, 15 and 17 belong to the third sphere, whereas the features 14, 16 and 18 belong to the fourth sphere. Similarly to the single-linkage cluster analysis the spheres method makes it possible to differentiate between high class agricultural distillates and other ones.

Summarizing, application of the electronic nose prototype allows differentiation of the samples of high class agricultural distillates from the remaining two quality classes. The results obtained with three methods of data analysis, principal component analysis, single-linkage cluster analysis, cluster analysis with spheres method are convergent. The authors believe that added concentration of volatile fraction components of the agricultural distillates could have the main influence on sample differentiation.

## 4. Summary

The aim of the conducted research was quality evaluation of agricultural distillates using an electronic nose instrument. An electronic nose prototype was designed and built in order to achieve that goal. The prototype was equipped with a set of six semiconductor sensors by FIGARO Co. Moreover, it contained the electronic circuits enabling conversion of voltage signals to digital form. Analytical signals were expressed in bits. Such an approach to signal processing allowed lowering the limit of detection of the semiconductor sensors by one order of magnitude [35]. Application of a set of thermostating modules in the electronic nose structure allowed for control of the gradient temperature characteristics of gas mixtures obtained via barbotage [39], thus providing stable and reproducible signals (93% of the obtained results were within the 5.8%–9.2% CV range). An additional advantage of the electronic nose prototype was the time of a single analysis, which together with data analysis lasted three minutes. In order to generate the gas phase of the agricultural distillates subjected to analysis with the electronic nose instrument, a barbotage process was employed consisting in extraction of volatile analytes from an alcohol-water matrix using a carrier gas.

## 5. Conclusions

The investigations proved an ability of differentiation between agricultural distillates of various quality classes. However, only for high quality class distillates identification of the class was feasible with all three methods of data analysis: principal component analysis, single-linkage cluster analysis and cluster analysis with spheres method. In the case of medium and low class distillates both cluster methods did not provide sufficient certainty of differentiation. The PCA method also only supplied some information allowing differentiation of medium and low class distillates. Such a situation is due to the still too high limit of detection of semiconductor sensors, and differing volatility and concentration levels of the components of agricultural distillates of medium and low quality, which do not make it possible to obtain high certainty via the aforementioned analytical methods. It was revealed that for this particular prototype of electronic nose the best (optimum) measurement conditions were as follows: sample volume 5 mL, volumetric flow rate of carrier gas 15 L/h, temperature of thermostat with sample subjected to barbotage 15 °C, time of sensor signal acquisition since bargotage process onset 60 s. Nevertheless, it seems that the electronic nose is a promising technique and there is high probability of its successful application to distillate quality evaluation.

## References

[1] Plutowska B., Biernacka P., Wardencki W., Brew J.I. (2010). Identification of volatile compounds in raw spirits of different organoleptic quality. J. Inst. Brew..

[2] Di Natale C., Davide F.A.M., D'Amico A., Nelli P., Groppelli S., Sberveglieri G. (1996). An electronic nose for the recognition of the vineyard of a red wine. Sens. Actuators B..

[3] Aleixandre M., Lozano J., Gutiérrez J., Sayago I., Fernández M.J., Horrillo M.C. (2008). Portable e-nose to classify different kinds of wine. Sens. Actuators B..

[4] Lozano J., Arroyo T., Santos J.P., Cabellos J.M., Horrillo M.C. (2008). Electronic nose for wine ageing detection. Sens. Actuators B..

[5] Lozano J., Fernández M.J., Fontecha J., Aleixandre M., Santos J.P., Sayago I., Arroyo T., Cabellos J.M., Gutiérrez F.J., Horrillo M.C. (2006). Wine classification with a zinc oxide SAW sensor array. Sens. Actuators B..

[6] Lozano J., Santos J.P., Arroyo T., Aznar M., Cabellos J.M., Gil M., Horrillo M.C. (2007). Correlating e-nose responses to wine sensorial descriptors and gas chromatography-mass spectrometry profiles using partial least squares regression analysis. Sens. Actuators B..

[7] Lozano J., Santos J.P., Gutiérrez J., Horrillo M.C. (2007). Comparative study of sampling systems combined with gas sensors for wine discrimination. Sens. Actuators B..

[8] Santos J.P., Arroyo T., Aleixandre M., Lozano J., Sayago I., García M., Fernández M.J., Arés L., Gutiérrez J., Cabellos J.M. (2004). A comparative study of sensor array and GC-MS: Application to Madrid wines characterization. Sens. Actuators B..

[9] Santos J.P., Lozano J., Aleixandre M., Sayago I., Fernández M.J., Arés L., Gutiérrez J., Horrillo M.C. (2004). Discrimination of different aromatic compounds in water, ethanol and wine with a thin film sensor array. Sens. Actuators B..

[10] Lozano J., Santos J.P., Horrillo M.C. (2005). Classification of white wine aromas with an electronic nose. Talanta.

[11] García M., Aleixandre M., Gutiérrez J., Horrillo M. (2006). Electronic nose for wine discrimination. Sens. Actuators B..

[12] Buratti S., Ballabio D., Benedetti S., Cosio M.S. (2007). Prediction of Italian red wine sensorial descriptors from electronic nose, electronic tongue and spectrophotometric measurements by means of Genetic Algorithm regression models. Food Chem..

[13] Brezmes J., Llobet E., Vilanova X., Saiz G., Correig X. (2000). Fruit ripeness monitoring using an Electronic Nose. Sens. Actuators B..

[14] Boilot P., Hines E.L., Gongora M.A., Folland R.S. (2003). Electronic noses inter-comparison, data fusion and sensor selection in discrimination of standard fruit solutions. Sens. Actuators B..

[15] Brezmes J., Llobet E., Vilanova X., Orts J., Saiz G., Correig X. (2001). Correlation between electronic nose signals and fruit quality indicators on shelf-life measurements with pinklady apples. Sens. Actuators B..

[16] Guadarrama A., Fernández J., Íñiguez M., Souto J., de Saja J. (2000). Array of conducting polymer sensors for the characterisation of wines. Anal. Chim. Acta..

[17] Guadarrama A., Ferna J., Souto J., de Saja J. (2001). Discrimination of wine aroma using an array of conducting polymer sensors in conjunction with solid-phase micro-extraction (SPME) technique. Sens. Actuators B..

[18] Lebrun M., Plotto A., Goodner K., Ducamp M.N., Baldwin E. (2008). Discrimination of mango fruit maturity by volatiles using the electronic nose and gas chromatography. Postharvest Biol. Technol..

[19] Martí M.P., Boqué R., Busto O., Guasch J. (2005). Electronic noses in the quality control of alcoholic beverages. Trends Anal. Chem..

[20] Dymerski T.M., Chmiel T.M., Wardencki W. (2011). Invited review article: An odor-sensing system-powerful technique for foodstuff studies. Rev. Sci. Instrum..

[21] Dymerski T., Chmiel T., Mostafa A., Śliwińska M., Wiśniewska P., Wardencki W., Namieśnik J., Górecki T. (2013). Botanical and geographical origin characterization of polish honeys by headspace SPME-GC×GC-TOFMS. Curr. Org. Chem..

[22] Plutowska B., Chmiel T., Dymerski T., Wardencki W. (2011). A headspace solid-phase microextraction method development and its application in the determination of volatiles in honeys by gas chromatography. Food Chem..

[23] Falqué E., Fernández E., Dubourdieu D. (2001). Differentiation of white wines by their aromatic index. Talanta.

[24] Gewu W. (1997). Identification of character impact odorants of different white wine varieties. J. Agric. Food Chem..

[25] Bauer-Christoph C., Christoph N., Aguilar-Cisneros B.O., Lopez M.G., Richling E., Rossmann A., Schreier P. (2003). Authentication of tequila by gas chromatography and stable isotope ratio analyses. Eur. Food Res. Technol..

[26] Cortés S., Gil L.M., Fernández E. (2005). Volatile composition of traditional and industrial Orujo spirits. Food Control.

[27] Ebeler S.E., Terrien M.B., Butzke C.E. (2000). Analysis of brandy aroma by solid-phase microextraction and liquid-liquid extraction. J. Sci. Food Agric..

[28] Diéguez S.C., Díaz L.D., Luisa M., de La Peña G., Gómez E.F. (2002). Variation of volatile organic acids in spirits during storage at low and room temperatures. Food Sci. Technol..

[29] Harynuk J., Górecki T. (2004). Comprehensive two-dimensional gas chromatography in stop-flow mode. J. Sep. Sci..

[30] Piñeiro Z., Palma M., Barroso C.G. (2004). Determination of terpenoids in wines by solid phase extraction and gas chromatography. Anal. Chim. Acta..

[31] Wardencki W., Chmiel T., Dymerski T., Biernacka P., Plutowska B. (2009). Application of gas chromatography, mass spectrometry and olfactometry for quality assessment of selected food products. Ecol. Chem. Eng. S..

[32] Aznar M., López R., Cacho J.F., Ferreira V. (2001). Identification and quantification of impact odorants of aged red wines from Rioja. GC-olfactometry, quantitative GC-MS, and odor evaluation of HPLC fractions. J. Agric. Food Chem..

[33] Callemien D., Dasnoy S., Collin S. (2006). Identification of a stale-beer-like odorant in extracts of naturally aged beer. J. Agric. Food Chem..

[34] Demyttenaere J.C.R., Dagher C., Sandra P., Kallithraka S., Verhé R., de Kimpe N. (2003). Flavour analysis of Greek white wine by solid-phase microextraction-capillary gas chromatography-mass spectrometry. J. Chromatogr. A..

[35] Dymerski T., Gębicki J., Wiśniewska P., Sliwińska M., Wardencki W., Namieśnik J. (2013). Application of the electronic nose technique to differentiation between model mixtures with COPD markers. Sensors.

[36] Maekawa T., Suzuki K., Takada T., Kobayashi T., Egashira M. (2001). Odor identification using a SnO_2_-based sensor array. Sens. Actuators B..

[37] Schaller E., Bosset J., Escher F. (1998). “Electronic Noses” and their application to food. Food Sci. Technol..

[38] Chueh H., Hatfield J. (2002). A real-time data acquisition system for a hand-held electronic nose (H_2_EN). Sens. Actuators B..

[39] Dymerski T., Wardencki W., Gębicki J., Fijało C., Świątoniowski B. Sposób oceny jakości destylatu rolniczego i urządzenie do oceny jakoŚci destylatów rolniczych.

